# What can we learn from HIV, COVID‐19 and mpox stigma to guide stigma‐informed pandemic preparedness?

**DOI:** 10.1002/jia2.26042

**Published:** 2022-12-05

**Authors:** Carmen H. Logie

**Affiliations:** ^1^ Factor‐Inwentash Faculty of Social Work University of Toronto Toronto Ontario Canada; ^2^ Women's College Research Institute Women's College Hospital Toronto Ontario Canada; ^3^ Centre for Gender & Sexual Health Equity Vancouver British Columbia Canada; ^4^ United Nations University Institute for Water Environment, & Health Hamilton Ontario Canada

1

The widespread emergence of stigma in the COVID‐19 pandemic resulted in comparisons with the devaluing, mistreatment and blame that people living with HIV have experienced for over four decades [1]. More recently, there were calls to learn from these epidemics to inform mpox responses [2]. Each of these epidemics reveals social processes of “othering,” whereby a person or group is labelled as “abnormal,” and of lower value than, one's “normal” self [3]. This othering fosters stigma that involves sequelae of social distancing and mistreatment, and ultimately reduces access to resources, power and opportunities. Stigma conceptual frameworks can inform pandemic preparedness to mitigate stigma in future pandemic responses.

A framework relevant to understanding similar processes of stigma targeting HIV, COVID‐19 and mpox includes three stigma archetypes: the “foreign” other, the “immoral” other and the “visibly” unwell. The first such archetype involves blaming a “foreign” other for epidemics, which is a historically recurring narrative [4]. In the case of the Bubonic plague, Jewish people were blamed in medieval Europe, and Chinese communities were blamed in 1900 in San Franciso [4]. Another illustrative example is syphilis, whereby countries blamed their neighbour: Russia referred to syphilis as a Polish disease, Poland referred to it as a German disease and France called it the “Neopolitan” disease [5]. With regard to HIV, Haitians were blamed for the emergence of HIV in the United States in the early 1980s, comprising one of the four “H” groups considered at elevated risk for HIV transmission and acquisition (the other “H”s’ included homosexuals, heroin users and haemophiliacs) [6]. Persons of Asian descent were blamed for the COVID‐19 pandemic across global contexts resulting in anti‐Asian racism. This stigma was exacerbated by the initial naming of COVID‐19 as the “Wuhan” or “China” virus by the media and key opinion leaders. Mpox, most often affecting persons living in Central and West Africa, is described as spreading to Europe or North America via travel of persons or animals originating from these regions. Indeed, two strains of mpox are labelled “West African” and “Congo Basin Central,” and the World Health Organization (WHO) recently renamed the virus from monkeypox to mpox and plans to rename the clades, as it recommends that illnesses are not named after a place or animal to reduce the potential for stigma.

The second stigma archetype is the “immoral” other. Gay, bisexual and other men who have sex with men (gbMSM) experience social, healthcare and often legal stigma across global contexts. They were blamed for the spread of both HIV [6] and recent mpox epidemics [2] through a focus on individual sexual practices; this individual focus can obscure larger social and historical mistreatment and exclusion that increase vulnerability to infectious diseases and constrain healthcare access. UNAIDS warned against public reporting on mpox that may “reinforce homophobic and racist stereotypes and exacerbate stigma” [7] and others have emphasized the importance of health messaging that avoids stereotyping Africans and gbMSM [2]. In the case of COVID‐19, persons not following pandemic restrictions were labelled “super spreaders” at the beginning of the pandemic, and more recently, there have been debates over whether stigmatizing unvaccinated persons as “immoral” is warranted [8]. Robust evidence, however, warns against *ever* using stigma as a public health tool, as it can cause further harm to marginalized communities [9].

Finally, the third stigma archetype targets persons with a “visible” health condition [3], aligning with the importance of considering concepts of peril, visibility and controllability [10]. Peril refers to how dangerous a stigmatized illness is perceived to be [10]. Visibility also shapes stigma experiences, whereby a person with a visible stigmatized condition manages tensions regarding being discredited and persons with an invisible stigma manage disclosure decisions (to who, when and how) [3]. Controllability refers to how much an individual is viewed as responsible for the stigmatized condition [10]. These three factors converge to shape how stigma is produced and perpetuated and can be applied to better understand HIV, COVID‐19 and mpox stigma. For instance, U = U messaging reduces HIV stigma by raising awareness of HIV as a treatable, non‐transmissible health condition, in turn reducing the *peril* surrounding HIV [11]. *Visible* signs of HIV‐associated health conditions, such as Kaposi's sarcoma, can increase HIV stigma exposure. Persons perceived as responsible for acquiring HIV through sexual practices experience greater stigma in contrast with persons constructed as “innocent victims,” such as haemophiliacs, as they are perceived to have been able to *control* their infection risk [2]. In the case of COVID‐19, fear of infection and perceived severity of COVID‐19—reflecting increased *peril*—were associated with higher stigma [12]. *Visible* ethno‐racial minorities, specifically persons of Asian descent, continue to experience mistreatment and even violence linked with blame for COVID‐19. As vaccination is linked with the *controllability* of COVID‐19, unvaccinated persons are blamed for causing harm to themselves and their communities. Others, however, situate COVID‐19 risk and vaccine hesitancy in larger social and structural contexts, including structural racism. For mpox, the existing diagnostic tools, vaccines and treatment—unlike in the early epidemics of HIV and COVID‐19 [2]—and its low mortality may reduce perceived *peril*. Yet, persons with mpox discuss the stigma and distress that are exacerbated with *visible* lesions. While the WHO and others describe mpox as *controllable* among gbMSM through altering sexual practices, which may increase stigma exposure, there are concerns raised regarding the lack of urgency in increasing global access to mpox testing, vaccination and treatment [2].

We can apply these lessons learned on stigma processes from HIV, COVID‐19 and mpox to guide stigma‐informed pandemic preparedness. In addition to assessing social processes of othering, and perceptions of peril, visibility and controllability, principles from the Health Stigma and Discrimination Framework [13] and intersectional stigma [14] can be applied by researchers, practitioners and policymakers to develop stigma‐informed pandemic responses, as detailed in Table 1. Guiding questions informed by the Health Stigma and Discrimination Framework [13], for instance, can assess underlying contextually specific stigma drivers (e.g. racist stereotypes) and facilitators (e.g. health policies) that shape stigma experiences and healthcare access among affected communities. Intersectional stigma approaches focus not only on identifying interlocking social categories (e.g. race and gender) linked with power and opportunity [14], but also on leveraging community strengths and solidarity.

**Table 1:** Guiding principles to address stigma in pandemic preparedness and pandemic responses with case examples from HIV, COVID‐19 and mpox

Guiding principle	Question examples for a stigma‐informed response	Case example: HIV	Case example: COVID‐19	Case example: Mpox
Examine underlying stigma drivers and facilitators	*Drivers*: What prejudice, stereotypes and judgement do affected communities experience? *Facilitators*: What are current: social inequities, occupational health and safety standards, and legal contexts for affected populations? What are relevant health policies and healthcare access?	*Drivers: —* prejudice, stereotypes and judgement experienced by affected communities (e.g. sex workers and men who have sex with men [MSM]) *Facilitators:—* criminalization (e.g. of sex work, LGBTQ persons, HIV non‐disclosure) *—* ethno‐racial, MSM and socio‐economic disparities in HIV	*Drivers:—* prejudice, stereotypes and judgement of Asian communities *—* social value of groups impacted by COVID‐19 *Facilitators: —* criminalization of public health responses *—* policies for accessing vaccines, testing and sick leave *—* occupational safety standards	*Drivers: —* prejudice, stereotypes and judgement experienced by MSM *—* community awareness of mpox *Facilitators*:—health policies regarding mpox testing, vaccination and treatment *—* health policies for sick leave and medical care access *—* ethno‐racial and MSM healthcare disparities
Assess peril, visibility and controllability	*Peril*: How dangerous is the infection considered? *Visibility*: Is this a visible or a concealable condition? *Controllability*: How responsible are persons perceived for acquiring the infection?	*Peril: —* extent of HIV treatment literacy *Visibility*:—signs of health conditions linked with HIV (e.g. Kaposi sarcoma) *Controllability: —* blame of key populations for HIV acquisition and transmission	*Peril: —* mortality and perceived severity of COVID‐19 *Visibility: —* visible Asian ethno‐racial minority persons targeted by anti‐Asian racism *Controllability: —* blame for becoming infected with COVID‐19	*Peril: —* severity of illness with mpox *Visibility: —* visibility of lesions and illness *Controllability: —* blame of persons with mpox, including MSM, for their infection
Identify community strengths	What social histories of solidarity, mutual support and collective care exist among affected communities? What community strengths can be leveraged in pandemic responses?	*—* LGBTQ community‐groups and AIDS service organizations that support affected communities *—* histories of HIV activism and mutual support	*—* mutual support or poverty alleviation networks for people who miss or lose employment due to COVID‐19 infection, quarantine and/or lockdowns *—* anti‐racist solidarity movements	*—* LGBTQ community‐groups and AIDS service organizations to support affected communities *—* community care networks to help persons access and locate vaccines and social/financial support if quarantined *—* global vaccine equity movements and advocacy for vaccine access in Africa

Activism, advocacy and collective care initiatives led by lesbian, gay, bisexual, transgender and queer communities in the HIV epidemic—and in mpox community‐based responses [2]—can reduce social isolation and experiences of perceived, anticipated and internalized stigma. Similarly, mutual aid and community‐driven care during COVID‐19 drew on underlying values of shared humanity and solidarity to challenge multiple forms of oppression [15]. Enriching our understanding of stigma processes that span health issues, populations and contexts can help us to meet the immediate needs of affected communities and embark on long‐term approaches to embed equity and social justice at the heart of pandemic preparedness and pandemic response.

## COMPETING INTERESTS

No competing interests are declared.

## FUNDING

CHL receives support for her programme of research from Canada Research Chairs, Canada Foundation for Innovation and the Ontario Ministry of Research and Innovation. Funders played no role in writing this manuscript.
